# ^64^Cu-DOTATATE PET/MRI for Detection of Activated Macrophages in Carotid Atherosclerotic Plaques: Studies in Patients Undergoing Endarterectomy

**DOI:** 10.1161/ATVBAHA.114.305067

**Published:** 2015-06-24

**Authors:** Sune Folke Pedersen, Benjamin Vikjær Sandholt, Sune Høgild Keller, Adam Espe Hansen, Andreas Ettrup Clemmensen, Henrik Sillesen, Liselotte Højgaard, Rasmus Sejersten Ripa, Andreas Kjær

**Affiliations:** From the Department of Clinical Physiology, Nuclear Medicine and PET (S.F.P., A.E.C., L.H., R.S.R., A.K., S.H.K., A.E.H.), Cluster for Molecular Imaging (S.F.P., A.E.C., L.H., R.S.R., A.K.), Department of Vascular Surgery (B.V.S., H.S.), Rigshospitalet and University of Copenhagen, Copenhagen, Denmark.

**Keywords:** atherosclerosis, endarterectomy, carotid, magnetic resonance imaging, positron-emission tomography

## Abstract

**Objective—:**

A feature of vulnerable atherosclerotic plaques of the carotid artery is high activity and abundance of lesion macrophages. There is consensus that this is of importance for plaque vulnerability, which may lead to clinical events, such as stroke and transient ischemic attack. We used positron emission tomography (PET) and the novel PET ligand [^64^Cu] [1,4,7,10-tetraazacyclododecane-*N*,*N*′,*N*″,*N*‴-tetraacetic acid]-*d*-Phe1,Tyr3-octreotate (^64^Cu-DOTATATE) to specifically target macrophages via the somatostatin receptor subtype-2 in vivo.

**Approach and Results—:**

Ten patients underwent simultaneous PET/MRI to measure ^64^Cu-DOTATATE uptake in carotid artery plaques before carotid endarterectomy. ^64^Cu-DOTATATE uptake was significantly higher in symptomatic plaque versus the contralateral carotid artery (*P*<0.001). Subsequently, a total of 62 plaque segments were assessed for gene expression of selected markers of plaque vulnerability using real-time quantitative polymerase chain reaction. These results were compared with in vivo ^64^Cu-DOTATATE uptake calculated as the mean standardized uptake value. Univariate analysis of real-time quantitative polymerase chain reaction and PET showed that cluster of differentiation 163 (CD163) and CD68 gene expression correlated significantly but weakly with mean standardized uptake value in scans performed 85 minutes post injection (*P*<0.001 and *P*=0.015, respectively). Subsequent multivariate analysis showed that CD163 correlated independently with ^64^Cu-DOTATATE uptake (*P*=0.031) whereas CD68 did not contribute significantly to the final model.

**Conclusions—:**

The novel PET tracer ^64^Cu-DOTATATE accumulates in atherosclerotic plaques of the carotid artery. CD163 gene expression correlated independently with ^64^Cu-DOTATATE uptake measured by real-time quantitative polymerase chain reaction in the final multivariate model, indicating that ^64^Cu-DOTATATE PET is detecting alternatively activated macrophages. This association could potentially improve noninvasive identification and characterization of vulnerable plaques.

## Abstract 2

Supplemental Digital Content is available in the text.

Carotid atherosclerosis is a major risk factor of stroke and transient ischemic attack. Randomized trials have shown that carotid endarterectomy significantly reduces the risk of recurrent stroke in patients with recent symptoms of transient ischemic attack or stroke and at least 50% stenosis of the relevant carotid artery.^1^ However, not all patients will benefit from the surgery because some will have stable plaques that are not prone to cause new thromboembolic lesions. In addition, many patients without significant carotid stenosis will experience recurrent stroke and could have benefited from endarterectomy.

A quest for new and more sensitive methods for in vivo identification of vulnerable atherosclerotic plaques is needed. Positron emission tomography (PET) for molecular imaging, typically in conjunction with anatomic imaging with computed tomography (CT), is one promising hybrid modality. The molecular tracer of choice has to date primarily been 2-[^18^F]-fluoro-2-deoxy-D-glucose (FDG). FDG is a glucose analogue that is taken up by high-glucose–using cells, where FDG is trapped by phosphorylation to allow for in vivo tissue glucose metabolism assessment. A large body of evidence has linked FDG uptake to the macrophage contents of high-risk atherosclerotic plaques.^2–4^ However, a major drawback of imaging atherosclerosis with FDG-PET is the lack of specificity of the tracer.

An alternative and potentially more specific target for imaging macrophages in the atherosclerotic plaque is the somatostatin receptor subtype-2, which is highly expressed by macrophages.^5^ This receptor can be imaged by PET using the ligand [1,4,7,10-tetraazacyclododecane-*N*,*N*′,*N*″,*N*‴-tetraacetic acid]-*d*-Phe1,Tyr3-octreotate (DOTATATE) labeled with a positron emitter. Two retrospective studies in patients with cancer investigated ^68^Ga-DOTATATE uptake in the coronary arteries^6^ and the large arteries.^7^ Both studies indicated an increased tracer uptake in atherosclerotic lesions. Interestingly, it was recently found that focal uptake of ^68^Ga-DOTATATE and FDG did not colocalize in a significant number of atherosclerotic lesions.^7^

We recently introduced DOTATATE labeled with ^64^Cu as an alternative to ^68^Ga labeling.^8^ ^64^Cu has a shorter positron range and longer half-life potentially improving spatial resolution and allowing for late image acquisition. In addition, we recently introduced the use of hybrid PET/MRI, which allows for more precise identification of the atherosclerotic plaque when compared with PET/CT.^9^

The aim of this study was for the first time to evaluate ^64^Cu-DOTATATE as an in vivo molecular tracer of atherosclerotic plaque activity. To do so, we compared in vivo tracer uptake with gene expression of molecular markers of macrophage load: cluster of differentiation 68 (CD68) as well as activated macrophages of the M1/M2 subsets: tumor necrosis factor-α (TNF-α) and CD163 in patients undergoing carotid endarterectomy using simultaneous PET/MRI in a prospective clinical trial. In addition, we aimed to establish the optimal time to wait from tracer injection to image acquisition.

## Materials and Methods

Materials and Methods are available in the online-only Data Supplement.

## Results

### Patient Population

Ten patients (5 men and 5 women, aged 53–73 years) with clinical symptoms of stroke or transient ischemic attack were enrolled in the study before clinically scheduled endarterectomy. One (symptomatic) plaque was recovered in toto from each patient and sectioned in 3-mm slices. This yielded different slice numbers per patient according to the physical size of each plaque specimen and came to a total of 10 plaques and 62 slices in all. Detailed patient characteristics can be seen in Table 1, and patient scan and surgery information are shown in Table 2.

**Table 1.**
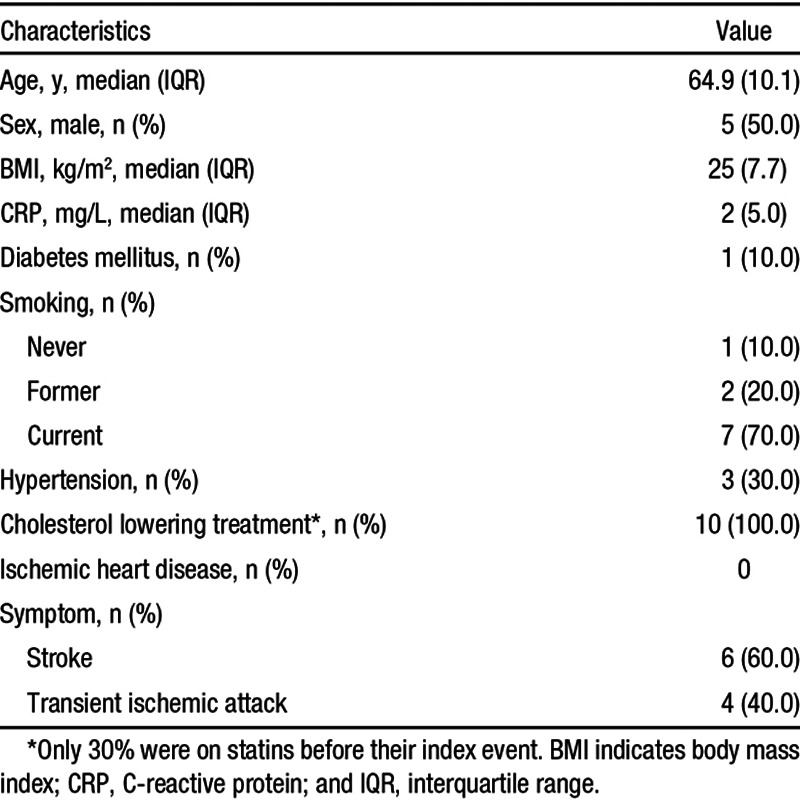
Patient Characteristics

**Table 2.**
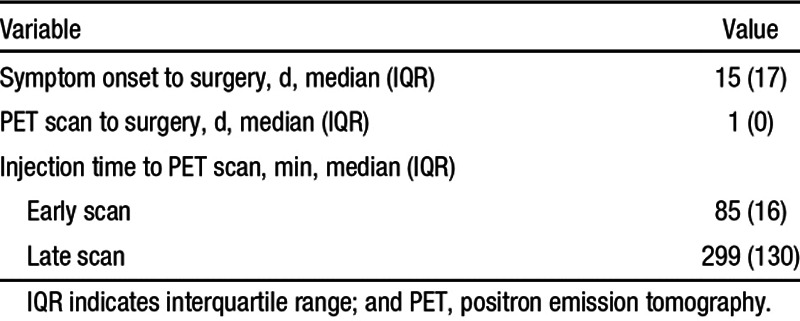
Patient Scan and Surgery Information

### Simultaneous PET/MRI of Carotid Atherosclerotic Plaques

**Figure 1.**
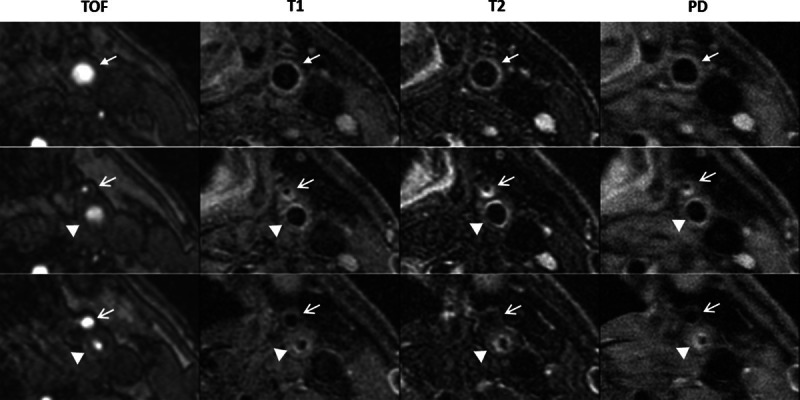
Multisequence MRI of the internal carotid artery at 3 different levels: first column; time-of-flight (TOF), second column; T1-weighted turbo-spin echo (T1), third column; T2-weighted turbo-spin echo (T2); and fourth column; proton density-weighted (PD). **Top**, Most caudal transaxial projection demonstrating *A. communis* (arrow). **Middle**, Intermediate transaxial projection demonstrating *C. interna* (arrowheads) and *C. externa* (open arrows). **Bottom**, Most cranial transaxial projection demonstrating *C. interna* (arrowheads) and *C. externa* (open arrows). Note the reduced blood flow on TOF in *C. externa* (**middle**) and *C. interna* (**bottom**). Plaque buildup can be seen in *C. externa* in T1, T2, and PD (**middle**) and in *C. interna* in T1, T2, and PD (**bottom**).

All patients underwent MRI for anatomic evaluation of carotid atherosclerosis simultaneously with collection of ^64^Cu-DOTATATE PET emission data. All 10 patients received an early scan and a total of 7 patients completed both an early and a late scan. Evidence of arterial wall thickening was seen both in the internal as well as the external carotid arteries in T1, T2, and PD-weighted imaging. These findings were matched by a pattern of stenotic lumen on time-of-flight–weighted imaging in the affected arteries (Figure 1). Plaque burden was determined using volumetric analysis: mm^3^ of index lesions (0.5±0.02; n=67) for all patients on a slice-by-slice basis. No correlation between MRI assessment of plaque burden and ^64^Cu-DOTATATE uptake determined by PET was found in univariate analysis (*P*=0.116).

**Figure 2.**
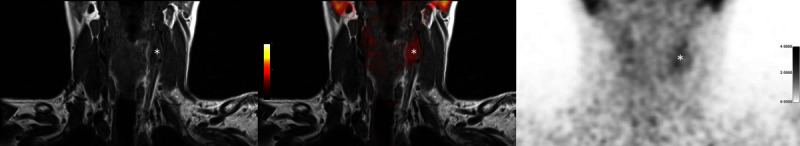
Coronal positron emission tomography (PET)/MRI of the neck region for visualization of the carotid arteries. **Left**, T1-weighted MR image showing atherosclerotic plaque of the left internal carotid artery marked with asterisk. **Middle**, **C**ombined PET/MRI of the same projection showing [^64^Cu] [1,4,7,10-tetraazacyclododecane-*N*,*N*′,*N*″,*N*‴-tetraacetic acid]-*d*-Phe1,Tyr3-octreotate (^64^Cu-DOTATATE) uptake in the plaque marked by asterisk. **Right**, Standalone PET image of the same projection showing ^64^Cu-DOTATATE distribution and left carotid artery plaque marked by asterisk.

**Figure 3.**
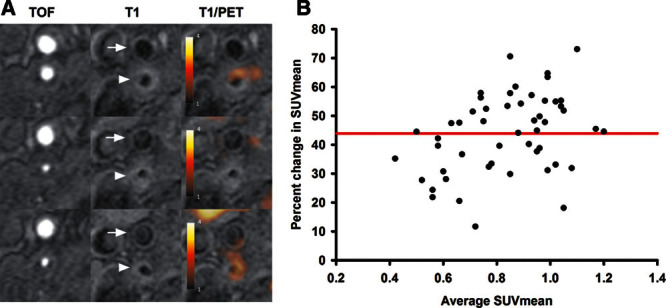
[^64^Cu] [1,4,7,10-tetraazacyclododecane-*N*,*N*′,*N*″,*N*‴-tetraacetic acid]-*d*-Phe1,Tyr3-octreotate (^64^Cu-DOTATATE) positron emission tomography (PET)/MRI visualization and raw data. **A**, PET/MRI from 1 patient at 3 consecutive levels: (arrow) *C. externa*; (arrowhead) *C. interna*; **top**, most caudal transaxial projection. **Left**, Time-of-flight weighted MRI; **middle**, T1-weighted MRI; **right**, combined T1-weighted MRI and PET. Heterogeneous ^64^Cu-DOTATATE uptake is seen in plaque of the *C. interna*: **top** and **bottom**, clear ^64^Cu-DOTATATE uptake (T1-weighted MRI/PET); **middle**, plaque with no uptake. **B**, Bland Altman comparison of standardized uptake value (SUV_mean_) from the early PET examination compared with SUV_mean_ from the late PET examination. Each black dot represents a 3-mm slice of the atherosclerotic plaque in the carotid artery. The red line represents the mean difference (in percent) between the 2 examinations.

**Figure 4.**
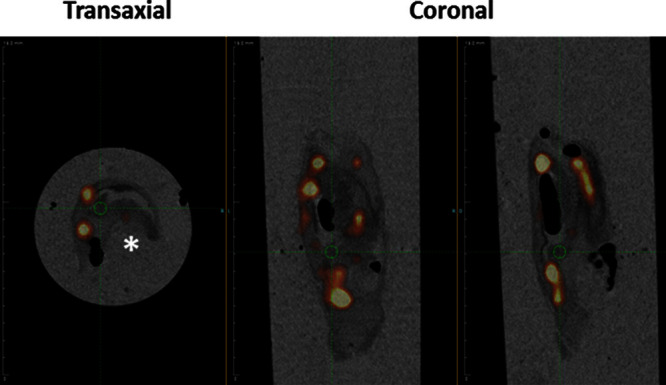
Ex vivo combined positron emission tomography/computed tomographic visualization of a plaque recovered from the internal carotid artery demonstrating heterogeneous [^64^Cu] [1,4,7,10-tetraazacyclododecane-*N*,*N*′,*N*″,*N*‴-tetraacetic acid]-*d*-Phe1,Tyr3-octreotate (^64^Cu-DOTATATE) uptake 23 hours post injection. **Left**, Transaxial projection of plaque exhibiting hot spots of ^64^Cu-DOTATATE accumulation. **Middle** and **right**, Coronal projections at 2 different levels. * indicates residual vessel lumen.

Examples of in vivo combined ^64^Cu-DOTATATE PET/MRI scans demonstrating tracer uptake in plaques of the internal carotid artery can be seen in Figures 2 and 3A. ^64^Cu-DOTATATE uptake was determined by mean standardized uptake value (SUV_mean_): early scan (1.18±0.03; n=61) and late scan (0.61±0.02; n=45). SUV_mean_ obtained early was significantly higher than SUV_mean_ obtained later, and a slice-by-slice comparison showed a mean difference of 43.9% (95% confidence interval, 40.1%–47.8%; n=45; Figure 3B). Also, we found broad limits of agreement between early and late PET scans (from 17.3%–70.6% of the early SUV_mean_). Finally, the SUV_mean_ values were significantly higher in the index lesion compared with the contralateral carotid artery (13.1% higher in index lesions; *P*<0.001, paired *t* test). Figure 3A shows representative images of ^64^Cu-DOTATATE PET/MRI scans of the internal carotid artery from a single patient at 2 different transaxial levels with heterogeneous and no uptake of ^64^Cu-DOTATATE, respectively. Overall ^64^Cu-DOTATATE uptake was heterogeneously distributed throughout the plaques. This finding was corroborated with the recovery of a single plaque that was visualized in toto using a preclinical PET/CT system to show the heterogeneity of tracer distribution ex vivo (Figure 4).

### Macrophage Detection in Carotid Atherosclerotic Plaques by ^64^Cu-DOTATATE PET, Immunohistochemistry, and Gene Expression Analysis

As for ^64^Cu-DOTATATE uptake, gene expression analysis from all slices and all patients demonstrated heterogeneity; note that gene expression data are log_2_ transformed: mean fold change in gene expression for CD163 showed (3.42±0.2; n=61), CD68 showed (3.4±0.2; n=61), cathepsin K showed (1.1±0.1; n=61), interleukin-18 showed (2.6±0.1; n=61), matrix metalloproteinase-9 showed (6.2±0.4; n=61), and TNF-α showed (1.4±0.1; n=62). Nonparametric testing (Spearman correlation matrix) demonstrated good interplaque correlation and strong statistical significance between the marker of activated macrophages (CD163) and the other molecular markers of plaque vulnerability (Table 3).

**Table 3.**
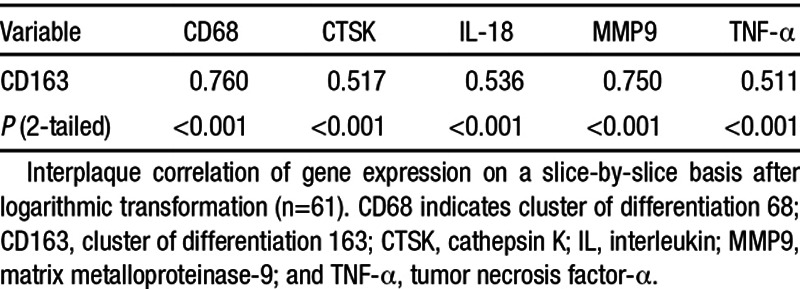
Spearman Correlation Coefficient Matrix of Gene Expression in Matched Plaque Slices

We used a mixed model to take into account the possible nonindependence of slices from the same patient. When entering 1 molecular marker (univariate), we found a weak but highly significant correlation between CD163 expression and ^64^Cu-DOTATATE uptake (*P*<0.001), as well as between CD68 expression and ^64^Cu-DOTATATE uptake (*P*=0.015). When entering both CD68 and CD163 into the mixed model, only CD163 remained significant (*P*=0.031). cathepsin K, interleukin-18, matrix metalloproteinase-9, and TNF-α were not significant when entered individually into the mixed model (Table 4). We also calculated target-to-background ratio values to compensate for background activity and performed the same statistical analyses with an identical outcome (data not shown).

**Table 4.**
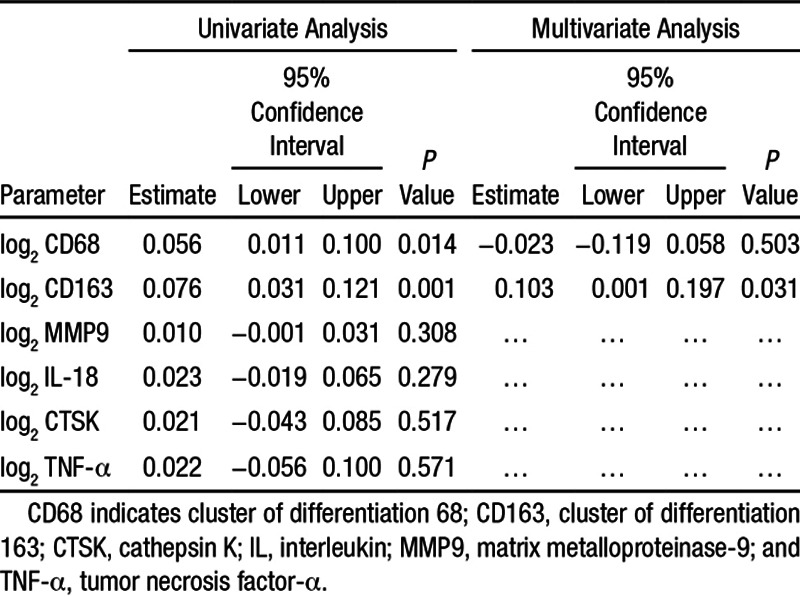
Results of the Linear Mixed Model (AR1) Estimates of Fixed Effects; Univariate and Multivariate Analyses With Mutual Adjustment

**Figure 5.**
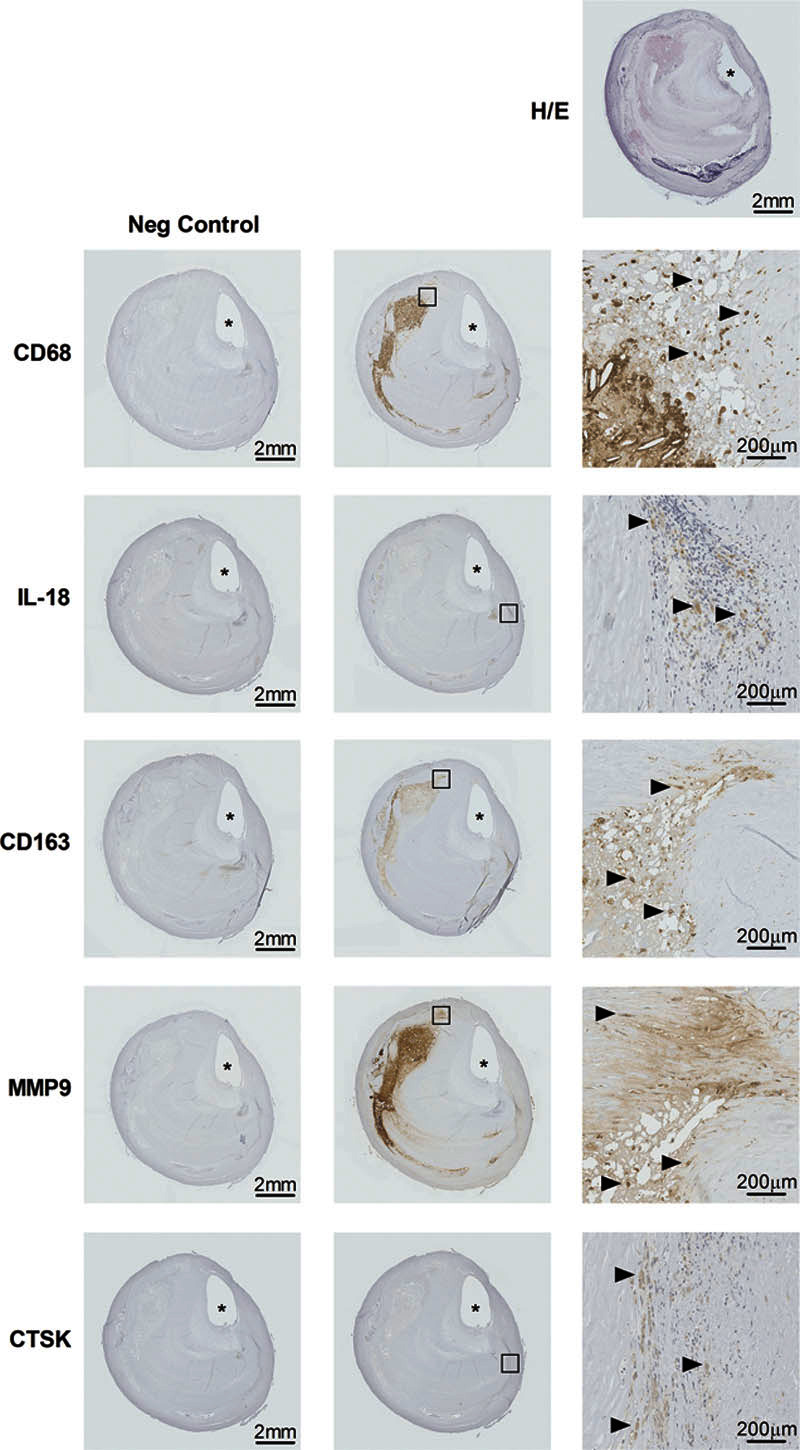
Case study showing histology and immunohistochemistry of an excised atherosclerotic plaque of the internal carotid artery immediately cranially to the bifurcature. **Top**, Hematoxylin/eosin (H/E) stain. All other rows: immunostaining, epitopes defined left of each row. **Left**, Negative control samples; **middle**, test samples; **right**, magnification from inserted boxes in middle panels. Scale bars provided in each panel in right lower corner. Arrowheads pointing right indicate positive immunostaining. * indicates residual arterial lumen. CD68 indicates cluster of differentiation 68; CD163, cluster of differentiation 163; CTSK, cathepsin K; IL-18, interleukin 18; and MMP9, matrix metalloproteinase-9.

A case study was subjected to immunohistochemical analysis. This confirmed the macrophage presence in the vicinity of and within the lipid core deep in the atheromateous plaque shown by specific CD68 and CD163 staining, which concomitantly colocalized with matrix metalloproteinase-9 expression (Figure 5). Furthermore, the presence of both the inflammatory cytokine interleukin-18 and cathepsin K was detected by immunohistochemistry (Figure 5).

## Discussion

In this study, we present the first results from simultaneous PET/MRI of human carotid plaques using the novel somatostatin receptor tracer ^64^Cu-DOTATATE. We found a highly heterogeneous intrapatient uptake within each atherosclerotic plaque. Interestingly, this uptake was significantly associated with biomarkers of macrophage load (CD68) and macrophage activation (CD163) in univariate analysis. This association seemed primarily driven by CD163 positive (CD163^+^) macrophages. This finding could have special interest because CD163^+^ macrophages are known to have an important role in plaques with hemorrhagic zones.^10^ In addition, we found evidence that time from tracer injection to PET acquisition is of paramount importance because tracer accumulation decreases almost 50% from our early scan to our late scan after correction for decay. Finally, we found no correlation between plaque burden and ^64^Cu-DOTATATE uptake.

### In Vivo Imaging

Only a few studies have used somatostatin receptor imaging in atherosclerosis.^6,7^ Both previous clinical studies used ^68^Ga-labeled DOTATATE. A preclinical study demonstrated colocalization of ^68^Ga-DOTATATE and macrophage-rich plaques by autoradiography in a mouse model.^11^ ^68^Ga has the advantage of being generator-eluted and thus has no need for an onsite cyclotron. However ^68^Ga has high maximum positron energy of 1.899 MeV witch translate into a high-positron range in water; 8.2 mm (maximum) and 2.9 mm (mean) and thus diminished spatial resolution. This is of vital importance for imaging small objects like carotid plaques. To circumvent this, we recently introduced ^64^Cu-labeled DOTATATE.^8^ ^64^Cu is a low-energy positron emitter (maximum positron energy, 0.653 MeV) with a positron range in water of 2.9 mm (maximum) and 0.64 mm (mean) that is comparable with that of ^18^F (≈1 mm), the isotope used in ^18^F-FDG PET. This is an essential advantage of ^64^Cu compared with ^68^Ga. In addition, ^64^Cu has a half-life of 12.7 hours giving the opportunity for delayed imaging. We found higher ^64^Cu-DOTATATE uptake in the index vessel compared with the contralateral carotid artery in our population; however, most included patients also had significant plaques in the contralateral vessel. We therefore suggest that it is reasonable to expect an even higher absolute difference in SUV_mean_ values between atherosclerotic versus healthy carotid arteries. Importantly, we found that plaque burden was not correlated with plaque ^64^Cu-DOTATATE uptake, indicating that plaque macrophage activity is not associated with plaque size per se. These results emphasize that PET has a promising role in molecular characterization of vulnerable plaques by providing in vivo information. We used hybrid PET/MRI instead of PET/CT in our study. MRI has superior soft tissue contrast allowing for better delineation of the carotid artery and atherosclerotic plaque when compared with CT.^9^ Finally, our PET/MRI system acquires the PET and MRI simultaneously allowing for perfect alignment between the 2 sets of images, when compared with the sequential acquisition in PET/CT where minor head movements can cause misalignment.

### CD68, CD163, and TNF-α

CD68 is a class D scavenger receptor and, although not exclusively expressed by macrophages, it is a widely used macrophage marker,^12,13^ which is why we use it as a surrogate measure of macrophage load. Several previous studies have shown a good correlation between CD68 and uptake of the glucose analogue FDG.^3,14–16^ Because FDG can be labeled with a positron emitter, this compound can be used for in vivo imaging. However, the major drawback of this imaging tracer is the low specificity of FDG limiting its use in assessing vulnerability of plaques.

CD163 is a hemoglobin scavenger receptor and macrophage-specific protein. It is upregulated in a subpopulation of alternatively activated M2 macrophages called hemorrhage-associated macrophages that are found in hemorrhagic zones of atherosclerotic plaques.^10,17^ A crucial role of hemorrhage-associated macrophages is to clear hemoglobin–haptoglobin complexes directly via the CD163 receptor and reduce oxidative stress, which subsequently mediates anti-inflammatory properties in vulnerable atherosclerotic plaques.^10,18,19^ Macrophages expressing CD163 have been detected in atherosclerotic plaques,^20^ and the soluble form of CD163 is associated with coronary atherosclerotic burden in the general population.^21^ The association between expression of CD163 and in vivo imaging of atherosclerotic plaques has not been investigated previously. However, a study of HIV infected patients found a correlation between plasma concentration of soluble CD163 and FDG uptake in the ascending aorta.^22^ Interestingly, a recent study into M1/M2 polarization of macrophage phenotypes in carotid plaques found that M2 macrophages are present in both symptomatic and asymptomatic plaques, whereas M1 macrophages are exclusive to symptomatic plaques.^23^

TNF-α is a cytokine involved in systemic inflammation and an important part of the acute phase reaction. It is produced primarily by activated macrophages in inflammation and considered a principal marker of M1 activation.^18^ Little recent work has been done on TNF-α expression in atherosclerotic lesions in humans; however, one investigation reported markedly raised (but nonsignificant) TNF-α RNA levels in symptomatic human endarterectomy specimens,^24^ and its role in systemic inflammatory disease is inarguable.^25,26^

Molecular markers of plaque vulnerability (cathepsin K, matrix metalloproteinase-9, and interleukin-18) that were previously found to be associated with FDG-uptake^3^ were not associated with ^64^Cu-DOTATATE uptake, highlighting how the 2 tracers image different biological processes using PET. Our finding substantiate that ^64^Cu-DOTATATE is correlated primarily with CD163 positive macrophages (CD163^+^) and only weaker with CD68 positive macrophages (CD68^+^) making ^64^Cu-DOTATATE uptake a predictor of (hemorrhage-associated macrophages) macrophage activity. Considering our selection of molecular M1/M2 macrophage-polarization markers (TNF-α/CD163), these results indicate that ^64^Cu-DOTATATE PET detects an M2-subset–driven macrophage response. Although we did find a good correlation between CD68 and CD163 gene expression (Table 3), this does not mean that these markers are equally coexpressed at the protein level because of post-transcriptional regulation mechanisms.^27^ Recently, immunohistochemistry was used to show that some overlap exist between CD163 and CD68 expression on the protein level, which indeed confirms our finding on the mRNA level; however, that study did not perform correlation analysis between these 2 markers either on mRNA or on protein levels.^17^ Using quantitative polymerase chain reaction as our primary end point meant that we could not perform a comparative analysis of ^64^Cu-DOTATATE uptake and sregional density (immunohistochemistry) of CD68/CD163 expression as the tissue slices were homogenized in toto in the mRNA isolation procedure. PET imaging with ^64^Cu-labeled DOTATATE is not equivalent to PET imaging with ^18^F-FDG as previously shown.^7^ The exact role of different macrophage subsets including CD163^+^ macrophages of the hemorrhage-associated phenotype in atherosclerosis needs further elucidation, and future studies will have to show whether ^64^Cu-DOTATATE has higher specificity than ^18^F-FDG for imaging vulnerability of plaques.

### Time of Imaging

We found only a moderate correlation between tracer accumulation at the early versus the later scan, and the significant association between tracer uptake and macrophage markers (CD68 and CD163) disappeared at the late time scan. In every single case, the SUV value decreased from early to late imaging indicating that binding to somatostatin receptor subtype-2 decreases over time. This is in contrast to PET imaging of atherosclerosis using ^18^F-FDG where imaging should be postponed to ≈3 hours after injection because FDG uptake in the plaques is either stable or increases, whereas FDG is cleared from the blood leading to improved target-to-background ratios.^28,29^ As FDG, in contrast to ^64^Cu-DOTATATE, is not bound to membrane-bound receptors but is continuously taken up and trapped by the cells, this discrepancy in optimal timing of imaging is not surprising. It could be speculated that instability of the ^64^Cu–DOTA complex in vivo^30–33^ and thereby increase in nonspecific background could be an explanation for the lack of correlation at the late time-point. However, because there was no increase in background activity observed, this explanation seems unlikely. Also, we recently published the first clinical PET study using the tracer ^64^Cu-DOTATATE in patients with neuroendocrine tumors. Here, we scanned patients 1, 3, and 24 hours post injection and did not see large liver accumulation or increase in blood-borne activity indicating that ^64^Cu-DOTATATE is indeed stable in humans for a long time.^8^ Also, as part of the approval by the Danish Health Authorities of ^64^Cu-DOTATATE, extensive stability studies were undertaken, demonstrating a shelf-life of at least 24 hours. Taken together, we find it most likely that decreasing signal-to-noise ratio over time explained by decrease in signal from receptor binding fully explains why early scans are superior. Based on our results, we therefore suggest that PET imaging of atherosclerosis with ^64^Cu-DOTATATE should be performed early, for example, at 80 minutes after injection.

### Limitations

Although our imaging method using ^64^Cu-DOTATATE seems promising, it is inherently challenged by low tracer uptake and a poor spatial resolution that could limit the usefulness. Nevertheless, the spatial resolution may not be a major obstacle, as it is at the same level as for FDG-PET, which increasingly seems to establish itself as an accepted method for plaque evaluation. However, only large clinical studies, ideally with head-to-head comparison with other methods, can establish the true clinical value of our method and its relation to other methods, for example, FDG-PET.

### Conclusions

In conclusion, we demonstrate the uptake of a novel PET tracer, ^64^Cu-DOTATATE, in human atherosclerotic plaques. We found a correlation between in vivo tracer uptake and ex vivo markers of activated macrophages. This association could potentially improve noninvasive identification of vulnerable plaques.

## Acknowledgments

The expertise and technical support of principal technicians Karin Stahr and Jakup Poulsen with positron emission tomography (PET)/MRI procedures and PET reconstructions are highly valued as is the expert statistical advice by Julie Lyng Forman.

## Sources of Funding

The unrestricted financial support from the Danish Heart Foundation, the Research Foundation of Rigshospitalet, the Danish Medical Research Council, and the John and Birthe Meyer Foundation is gratefully acknowledged. The PET/MRI scanner was donated by The John and Birthe Meyer Foundation.

## Disclosures

None

## Supplementary Material

## Significance

Macrophages express the somatostatin receptor subtype-2. By targeting this receptor using the ligand [64Cu] [1,4,7,10-tetraazacyclododecane-*N*,*N*′,*N*″,*N*‴-tetraacetic acid]-d-Phe1,Tyr3-octreotate (^64^Cu-DOTATATE), macrophages can be imaged noninvasively using positron emission tomography (PET). We hypothesized that in vivo ^64^Cu-DOTATATE uptake quantified by PET would correlate with ex vivo markers of macrophage infiltration. Atherosclerotic plaques of the internal carotid artery were imaged using ^64^Cu-DOTATATE PET. Subsequently, plaques recovered by carotid endarterectomy were analyzed using real-time quantitative polymerase chain reaction to assess molecular markers of macrophage infiltration and activity. We found a correlation between ^64^Cu-DOTATATE PET and gene expression of the hemoglobin/haptoglobin scavenger receptor CD163 by macrophages, also considered a marker of macrophage activation. Our work thus demonstrates that ^64^Cu-DOTATATE PET can be used to detect a hemorrhage-associated subpopulation of macrophages (CD163^+^) in vivo in atherosclerotic plaques of the internal carotid arteries. This finding has potential clinical implications for noninvasive identification of vulnerable plaques.
