# Supplementary material for: 64Cu-DOTATATE PET/MRI for Detection of Activated Macrophages in Carotid Atherosclerotic Plaques: Studies in Patients Undergoing Endarterectomy
Source: Arterioscler Thromb Vasc Biol. 2015 Jun 24;35(7):1696–703. doi: 10.1161/ATVBAHA.114.305067 (PMC4479665; doi:10.1161/ATVBAHA.114.305067)
Supplement: Supplementary file 1 [file atv-35-1696-s001.pdf]

Supplemental material

Study design

All patients referred to our institution for carotid endarterectomy were screened for inclusion. Inclusion criteria were significant carotid stenosis with recent cerebrovascular symptoms; stroke, amaurosis fugax or TIA. Exclusion criteria were age <50 years, current infection, pregnancy, renal insufficiency, or severe claustrophobia. All patients underwent two simultaneous PET/MR procedures after one injection of a mean of 154 MBq of ^{64}Cu-DOTATATE. An early PET/MR was performed a median of 85 minutes (range: 56-90 min) after injection and the late PET/MR was performed a median of 299 minutes (range: 265-428 min) after injection. Carotid endarterectomy was performed the following day using standard clinical procedure. The plaque specimens were conserved for later gene expression analysis. The study was approved by the Regional Scientific Ethical Committee (protocol H-4-2012-064) and all patients received oral and written information about the study and signed an informed consent prior to inclusion.

In-vivo PET/MR acquisition

Simultaneous PET/MR was performed on the Biograph mMR[1] (Siemens Healthcare, Erlangen, Germany) using the mMR standard PET compatible dedicated head and neck coil (Siemens Healthcare). Patients were placed in the scanner in the supine position and scout images were obtained for localization of the carotid arteries. An mMR standard Dixon water-fat MR sequence was then recorded and segmented (into air, lung tissue, soft tissue and fat) for MR-based PET attenuation correction[2]. PET acquisition was started along with the Dixon MR sequence. Acquisition time was 10 min in three-dimensional list-mode (scanner default list mode recording) in one bed position. A 3D time-of-flight (TOF) magnetic resonance angiography sequence was then performed to obtain lumen contours. MR parameters for 3D-TOF were: TR/TE 21/3.6 ms, voxel size $0.8\times0.5\times1.0$ mm^3. Next, a stack of 14 axial 2D turbo spin echo dark blood slices (thickness 2.2 mm, gap 0.88 mm, field of view 150 mm) with fat suppression and proton density, T1 and T2 weightings was placed using the 3D-TOF angiography. Images were centered at the stenotic area. MR parameters were: T1-weighted: TR/TE=1,060/26 ms, voxel size 0.6×0.6 mm^2. T2-weighted: TR/TE=2,500/76 ms, voxel size 0.6×0.6 mm^2. Proton density-weighted: TR/TE=2,400/16 ms, voxel size 0.4×0.4 mm^2. For detailed MR parameters of 3D TOF, T1, T2 and proton density-weighted sequences see supplementary material. On average, the total examination time was below 30 minutes.

MR quantification

The carorid artery was analyzed slice by slice on transaxial images in every subject guided by T1, T2, TOF and PD images by the same operator. Free-hand regions of interest were drawn to include vessel wall, plaque and lumen. Next vessel lumen was determined in an identical manner and finally the two volumes were subtracted to give a measure of plaque burden (mm^3) on a slice by slice basis.

PET image reconstruction

The in vivo PET data was reconstructed using ordered-subsets expectation maximization iterative 3-dimensional reconstruction (OSEM 3D). The reconstruction was done using 6 iterations, 21 subsets and zoom = 2.0 with MR based attenuation correction yielding 127 image slices each 512x512 voxels of size: 0.70x0.70x2.03mm^3 which were filtered post-reconstruction with a 2 mm full width half maximum Gaussian filter. The µ-maps for attenuation correction of the PET data were generated on the basis of the Dixon water-fat MR sequence[2] which is the standard MR attenuation correction for the Biograph mMR scanner. The segmentation and generation of the µ-maps from the Dixon sequence correction was done fully automatically by the scanner software.

PET quantification
Anatomical co-registration of MR and PET was carefully checked by matching anatomical landmarks like salivary glands. The carotid artery was analyzed slice by slice in every subject. The PET data was fused with the simultaneously acquired axial MR images (T1, T2, and proton density weighted). A free-hand region of interest (ROI) was drawn to include plaque, vessel wall and lumen starting at the carotid bifurcation proceeding caudally and cranially (not including the external carotid artery) to include the entire plaque. The mean standardized uptake value (SUV_{mean}) corrected for injected dose, patient weight and time to acquisition were calculated for each region of interest. For calculation of target-to-background (TBR) values the, SUV_{mean} for each slice was normalized to blood pool activity (SUV_{mean}) obtained from a ROI placed in the ipsilateral jugular vein resulting in TBR values for subsequent calculations.

Ex vivo PET/CT
For the *ex vivo* acquisition, one extirpated plaque underwent PET/CT imaging 23 hours after injection of ^{64}Cu-DOTATATE using a Siemens Inveon MM PET/CT small animal scanner (Siemens Molecular Imaging, Knoxville, USA). The specimen was PET-scanned for 90 minutes with a 350-650 keV energy window and 3.432 ns coincidence window. The PET list mode data was reconstructed using an OP-MAP iterative algorithm, applying 18 iterations and 2 subsets, with a requested resolution of 1.0 mm. The CT scan was done using x-ray voltage and current of 50 kVp and 500 mA, respectively. Projections were acquired over 360 degrees using 720 steps, with an exposure time of 2 seconds and 1 second of settle time. The CCD detector was using 4096 by 4032 pixels and a binning factor of 2. The CT projections were reconstructed into an image using filtered back-projection resulting in an isotropic voxel size of 21.7 µm. The images were co-registered and processed using Siemens Inveon Research Workplace version 4.1 (Siemens Molecular Imaging, Knoxville, USA).

Tissue RNA extraction
Tissue conservation and cDNA production for real-time qPCR analysis was performed as earlier described[3-5]. Briefly; plaque tissue secured by carotid endarterectomy was sliced immediately at bedside in the operating theater into 3 mm slices caudally (common carotid artery) and cranially (internal carotid artery) oriented from the vessel bifurcature. A custom made knife with blades spaced evenly at 3 mm intervals was used to ensure consistency of slice thickness. The slices corresponded to the PET/MR imaging modality and were immediately conserved in RNAlater® (cat. # AM7021, Ambion (Europe) Limited, Cambridgeshire, United Kingdom) in 1.5 mL PCR grade Eppendorf tubes for subsequent RNA extraction (n=61 slices). One plaque was conserved *in toto* for *ex vivo* scanning prior to slicing and RNA extraction. Additionally a piece of superior thyroid artery was secured as reference tissue from every patient. Subsequently tissue was stored at 5°C for 24 hours before RNAlater® was drained and the tissue transferred to -80°C storage.

RNA extraction was performed in a specially assigned RNA laboratory and procedures were executed in a fume cupboard. Each tissue slice and reference tissue was freeze fractured *in toto* using a cryoPREP™ impactor (Covaris Europe, Brighton, UK) and TRI Reagent® (cat. # TR118, Molecular Research Center Inc., Cincinnati, USA) was used for RNA isolation in a protocol modified from the manufacturer's instructions to accommodate freeze fracture: Each sample was transferred from Eppendorf tubes into PCR grade tissue bags (cat. # TT1XT, Covaris Inc., Europe, Brighton, UK) cooled in liquid Nitrogen and freeze fractured using the impactor. The pulverized sample was transferred to fresh 1.5 mL PCR grade Eppendorf tubes and 1000 µL TRI Reagent® added along with 100 µL of BCP (cat. # BP151, Molecular Research Center Inc., Cincinnati, USA). The samples were stirred vigorously using a vortex mixer for 15 seconds and left to incubate at room temperature for 15 minutes. Subsequently the samples were centrifuged at 12.000 *g* for 15 minutes at 4°C leaving an upper aqueous phase containing RNA, an interphase containing DNA and a lower (red) phase containing protein. The upper phase (450 µL) was carefully transferred to a fresh 1.5 mL Eppendorf tube and an equal volume (450 µL) of isopropanol was added for RNA

precipitation. The samples were mixed gently by hand and left to incubate for 10 minutes at room temperature. Centrifugation at 12.000 *g* at 4°C for 8 minutes was followed by careful removal of the supernatant leaving an RNA pellet. The pellet was then washed 2 times using 1 mL of RNase-free 75% ethanol alternating with centrifugation at 7.500 *g* at 4°C for 5 minutes before a final centrifugation at 13.000 *g* at 4°C for another 5 minutes where the remaining supernatant could be removed and pellet left to air dry for a minimum of 20 minutes. Finally 20 µL of molecular grade water (cat. # 2900136, 5-Prime GmbH, Hamburg, Germany) was added and the RNA reconstituted by vigorous mixing for 15 seconds using a vortex mixer. The RNA was stored at -80°C until further processing.

RNA quality and concentration
The RNA quality was assessed using the Agilent 2100 Bioanalyzer and associated reagents (cat. # G2938-80023, Agilent Technologies Inc., Santa Clara, CA, USA) to assign RNA integrity values (RINs)[6]. The system utilizes chip-based microfluidic technology providing electrophoretic separation in an automated and reproducible manner of up to 12 samples pr chip. RNA samples (1 µL pr sample) were separated in the channels of the chip according to molecular weight and detected by laser induced fluorescence detection of an added dye. Electropherograms for each sample was constructed for visualization of RNA quality. The “Agilent RNA 6000 Nano Assay Protocol – Edition April 2007” was used for preparing and assigning RIN values to all samples. In preparation for analysis 2 µL of samples and 1.2 µL of ladder (cat. # 5067-1529, Agilent Technologies Inc., Santa Clara, CA, USA) were denatured at 70°C for 2 minutes.

Briefly: RNA 6000 Nano gel matrix (550 µL) was pipetted into a spin filter and centrifuged at 1500 *g* for 10 minutes at room temperature and divided into 65 µL aliquots. Concentrated RNA 6000 Nano dye was stirred for 10 seconds and 1 µL added to the 65 µL of the RNA 6000 Nano gel matrix. The gel-dye mix was then vigorously stirred using a vortex mixer and subsequently centrifuged at 13.000 *g* for 10 minutes at room temperature. A 6000 Nano chip (cat. # 5067-1511, Agilent Technologies Inc., Santa Clara, CA, USA) for determination of total eukaryotic RNA was placed in the chip priming station and loaded with gel-dye mix for priming of the chip using the plunger system of the priming station. Once primed, the sample and ladder wells were all loaded with 5 µL of RNA 6000 Nano Marker whereas 1 µL of sample and 1µL of ladder was added to the wells marked “sample” and “ladder” respectively. The chip was stirred in a custom vortexer for 1 minute at 2400 rpm and the analyses were performed on the Bioanalyzer immediately thereafter.

RNA concentrations from the isolation procedure were measured using the Nanodrop 1000 spectrophotometer (Thermo Scientific, Waltham, MA, USA) prior to dilution for the cDNA synthesis step.

cDNA synthesis
Using the AffinityScript™ QPCR cDNA Synthesis Kit (cat. # 600559 Stratagene, La Jolla, CA, USA), 0.3 ng of total RNA was reverse transcribed to cDNA for subsequent real-time qPCR analysis of whole-slice gene expression. In short: A mixture containing 7 µL (0.3 ng) RNA, 10 µL first strand master mix (2x), 2.45 µL Oligo (dT) primer (100 ng/µL), 0.55 µl random primer (100 ng/µL) and 1 µL of Stratascript™ RT/RNase Block Enzyme Mixture were prepared and run on a Mastercycler gradient RT-PCR machine (Eppendorf AG, Hamburg, Germany) utilizing a program of 5 minutes of primer annealing at 25 °C, 15 minutes of cDNA synthesis at 42 °C and finally termination for 5 minutes at 95 °C. Subsequently cDNA was stored at -20 °C until qPCR analysis.

Quantitative real-time PCR
Optimal housekeeping genes were identified as described previously[3-5]. Briefly; we tested both reference and diseased tissue, using a commercially available panel of 12 housekeeping genes (cat. # A101, Version 1.3 TATAA Biocenter AB, Göteborg Sweden) and found 60S acidic ribosomal protein P0 (RPLP, gene ID: NM_001002) and peptidylprolyl isomerase A (PPIA, gene ID:

NM_021130) to be optimal using the geNorm algorithm embedded in the qBase^{PLUS} software (Biogazelle NV, Zwijnaarde, Belgium)[7]. Taqman® based gene expression assays were designed using Beacon Designer™ 7.90 (PREMIER Biosoft, Palo Alto, CA, USA) and analyzed using Mx3000P® or Mx3005P™ real-time PCR systems (Stratagene, La Jolla, CA, USA). Five genes of interest; cluster of differentiation 68 (CD68, gene ID: NM_001251) cluster of differentiation 163 (CD163, gene ID: NM_004244), matrix metalloproteinase 9 (MMP9, gene ID: NM_004994), interleukin 18 (IL-18, gene ID: NM_001562),cathepsin K (CTSK, gene ID: NM_000396) and tumor necrosis factor α (TNFα, gene ID: NM_000594.3) were analyzed. The thermal profile of the TaqMan® assays consisted of an initial denaturization step at 95°C for 10 minutes followed by 40 cycles of denaturization at 95°C for 15 seconds and finally combined annealing and elongation for one minute at 60°C. Gene expression data analysis was performed in qBase^{PLUS} incorporating a modified delta-delta-Cq model ($2^{\Delta\Delta Cq}$) and multiple reference genes[8, 9]. Inter-run calibration (three different samples replicated on every plate in each run) was used to eliminate plate-to-plate variation between runs. For assay and analysis details, see Tables I and II. All primers and Taqman® probes were purchased at Sigma (Sigma-Aldrich, St. Louis, MO, USA).

Histology

Recovered carotid plaque specimens were fixed in 10% neutrally buffered formalin for 24 hours and subsequently processed and paraffin embedded. A representative plaque was sectioned (4 µm), transferred to slides, deparaffinized and stained with Mayer's hematoxylin and eosin (cat. # 860213 and 854653, Region H Apothecary, Copenhagen, Denmark) before being dehydrated using ethanol and finally mounted with pertex.

Immunohistochemistry

Immunohistochemistry was performed to assess markers of vulnerability using mouse monoclonal antibodies against macrophages (CD68; dilution 1:100, clone PG-M1, Dako, Glostrup, Denmark [cat. # M087629-2] and CD163; dilution 1:100, clone EDHu-1, AbD Serotec, Kidlington, UK [cat. # MCA1853T]), a cytokine (IL-18; dilution 1:75, clone H44, abcam®, Cambridge, UK [cat. # ab140834]) and both monoclonal mouse and rabbit antibodies against markers of proteolysis (CTSK; dilution 1:150, clone ab66237, abcam®, Cambridge, UK [cat. # ab66237] and MMP9; dilution 1:150, clone EP1255Y, abcam®, Cambridge, UK [cat. # ab137867] respectively). Briefly: 4 µm sections was mounted on glass slides and placed at 40°C overnight. Next day, the temperature was increased for one hour to 60°C and the slides deparaffinized in shifts of xylen for 15 minutes followed by multiple shifts of ethanol in decreasing concentrations advancing the sections to demineralized water. Epitope demasking was performed using heat-induced epitope retrieval (HIER) in citrate buffer pH 6.0 for 15 minutes in a microwave oven followed by 30 minutes rest at room temperature and then immersion in phosphate-buffered saline (PBS) containing 0.1% Tween 20 (cat. # P1379-25mL, Sigma-Aldrich, St. Louis, MO, USA) for five minutes. Subsequent reactions all took place at room temperature. After 10 minutes of immersion in PBS, the slides were transferred to humidity chambers where each section was covered with peroxidase blocker (cat.# S2023, Dako, Glostrup, Denmark) for eight minutes and then rinsed with three shifts of PBS every two minutes. The sections were blocked using 2% bovine serum albumin (cat. # A7906-100g, Sigma-Aldrich, St. Louis, MO, USA) for 10 minutes followed by primary antibody diluted in 2% bovine serum albumin for samples and positive control tissue, whereas species-matched FLEX control (cat. # IS600 [rabbit] / IS750 [mouse], Dako, Glostrup, Denmark) was added to negative control samples. The samples were then incubated for one hour followed by rinse in three shifts of PBS every two minutes. Species matched secondary horse-radish peroxidase conjugated antibody was now added to the samples; EnVision FLEX (cat. # K4003 [rabbit] / K4001 [mouse], Dako, Glostrup, Denmark) which were left to incubate for another 40 minutes followed by rinse in two shifts of PBS for five minutes. The samples were developed using 3,3'-Diaminobenzidine (DAB) for 10 minutes (cat. # K3468, Dako, Glostrup, Denmark) followed by rinse in three shifts of PBS. The samples were transferred to soak in demineralized water for five minutes, counterstained by two

minutes of Mayer’s hematoxylin, soaked for five minutes in demineralized water, before being dehydrated in ethanol and finally mounted using pertex.

Slides were scanned using an Axio Scan.Z1 slide scanner (Carl Zeiss Microscopy GmbH, Jena, Germany) and subsequent image preparation was performed using a combination of Photoshop CS6 (Adobe Systems, San Jose, CA, USA) and Microsoft Powerpoint 2010 (Microsoft Corporation, Redmond, WA, USA).

Statistical analysis

Data was analyzed using a linear mixed model adjusting for serial correlation (AR1) predefined in repeated measurements from the same patient. Effects of explanatory variables (CD163, CD68, CTSK, IL-18, MMP9 and TNFα) were tested in univariate analyses. Signficiant explanatory variables (CD163, CD68) were thereafter included in a multivariate analysis. Gene expression and MR data are presented as mean ± SEM. Analyses were performed using SPSS 20 (IBM Corporation, Armonk, New York, USA) while Graphpad Prism 5 (Graphpad software Inc, La Jolla, CA, US) was used for illustrations. Normal distribution assumptions were tested using the Kolmogorov-Smirnov test. Furthermore in vivo PET data and gene expression analyses were compared using the Spearman correlation coefficient as appropriate. Early and late PET data were compared using paired samples t-test and Bland Altman statistics. Logarithmic transformation (log_2)of the gene expression data was applied to obtain normal distribution. $P<0.05$ was considered statistical significant.

References

1 Delso G, Furst S, Jakoby B, Ladebeck R, Ganter C, Nekolla SG, Schwaiger M, Ziegler SI. Performance measurements of the Siemens mMR integrated whole-body PET/MR scanner. J Nucl Med 2011 December;52:1914-22.

2 Martinez-Moller A, Souvatzoglou M, Delso G, Bundschuh RA, Chefd'hotel C, Ziegler SI, Navab N, Schwaiger M, Nekolla SG. Tissue classification as a potential approach for attenuation correction in whole-body PET/MRI: evaluation with PET/CT data. J Nucl Med 2009 April;50:520-6.

3 Pedersen SF, Graebe M, Hag AM, Hojgaard L, Sillesen H, Kjaer A. (18)F-FDG imaging of human atherosclerotic carotid plaques reflects gene expression of the key hypoxia marker HIF-1alpha. Am J Nucl Med Mol Imaging 2013;3:384-92.

4 Pedersen SF, Graebe M, Fisker Hag AM, Hojgaard L, Sillesen H, Kjaer A. Gene expression and 18FDG uptake in atherosclerotic carotid plaques. Nucl Med Commun 2010 May;31:423-9.

5 Pedersen SF, Graebe M, Hag AM, Hoejgaard L, Sillesen H, Kjaer A. Microvessel density but not neoangiogenesis is associated with 18F-FDG uptake in human atherosclerotic carotid plaques. Mol Imaging Biol 2012 June;14:384-92.

6 Schroeder A, Mueller O, Stocker S, Salowsky R, Leiber M, Gassmann M, Lightfoot S, Menzel W, Granzow M, Ragg T. The RIN: an RNA integrity number for assigning integrity values to RNA measurements. BMC Mol Biol 2006;7:3.

7 Vandesompele J, De PK, Pattyn F, Poppe B, Van RN, De PA, Speleman F. Accurate normalization of real-time quantitative RT-PCR data by geometric averaging of multiple internal control genes. Genome Biol 2002 June 18;3:RESEARCH0034.

8 Hellemans J, Mortier G, De PA, Speleman F, Vandesompele J. qBase relative quantification framework and software for management and automated analysis of real-time quantitative PCR data. Genome Biol 2007;8:R19.

9 Livak KJ, Schmittgen TD. Analysis of relative gene expression data using real-time quantitative PCR and the 2(-Delta Delta C(T)) Method. Methods 2001 December;25:402-8.

3D-TOF:SIEMENS: fl_tof

Routine
Slab group 1
Slabs 2
Dist. factor -19.23 %
Position L0.7 A26.1 F0.1
Orientation Transversal
Phase enc. dir. R >> L
Rotation 90.00 deg
Phase oversampling 0 %
Slice oversampling 23.1 %
Slices Per Slab 52
FoV read 200 mm
FoV phase 75.0 %
Slice thickness 1.00 mm
TR 21 ms
TE 3.60 ms
Averages 1
Concatenations 2
Filter Distortion Corr.(2D), Elliptical filter
Coil elements HEA;HEP;NEA;NEP
Contrast
TD 0.000 ms
MTC Off
Flip Angle 25 deg
Fat suppr. None
Water suppr. None
- -
Averaging mode Short term
Reconstruction Magnitude
Measurements 1
Resolution
Base resolution 384
Phase resolution 65 %
Slice resolution 63 %
Phase partial Fourier Off
Slice partial Fourier 6/8
Interpolation Off
- -
PAT mode GRAPPA
Accel. factor PE 2
Ref. lines PE 24
Accel. factor 3D 1
Matrix Coil Mode Auto (Triple)
Reference scan mode Integrated
- -
Image Filter Off
Distortion Corr. On

Mode 2D
Unfiltered images On
Prescan Normalize Off
Normalize Off
B1 filter Off
Raw filter Off
Elliptical filter On
Mode Inplane
POCS Off
Geometry
Multi-slice mode Sequential
Series Descending
- -
- - - - - - - - - - - -
Special sat. Tracking H
Gap 10 mm
Thickness 40 mm
- -
- - - - - - - - - - - -
Set-n-Go Protocol Off
Table Position H
Table Position 0 mm
Inline Composing Off
Sequence
Introduction On
Dimension 3D
Elliptical scanning Off
Asymmetric echo Allowed
Contrasts 1
Bandwidth 250 Hz/Px
Flow comp. Yes
- -
- - - - - - - - - - - -
Gradient mode Fast
RF spoiling On
651/+

T1-weighted:

SIEMENS: tse
Routine
Slice group 1
Slices 14
Dist. factor 40 %
Position Isocenter
Orientation Transversal
Phase enc. dir. R >> L
Rotation 90.00 deg
Phase oversampling 0 %
FoV read 150 mm
FoV phase 100.0 %
Slice thickness 2.2 mm
TR 1060 ms
TE 26 ms
Averages 2
Concatenations 2
Filter Distortion Corr.(2D), Elliptical filter
Coil elements HEA;HEP;NEA;NEP
Contrast
TD 0.0 ms
MTC Off
Magn. preparation None
Flip Angle 180 deg
Fat suppr. SPAIR
Fat sat. mode Strong
Water suppr. None
Restore magn. Off
- -
Averaging mode Short term
Reconstruction Magnitude
Measurements 1
Multiple series Each measurement
Resolution
Base resolution 256
Phase resolution 100 %
Phase partial Fourier Off
Trajectory Cartesian
Interpolation Off
- -
PAT mode None
Matrix Coil Mode Auto (CP)
- -
Image Filter Off
Distortion Corr. On
Mode 2D
Unfiltered images On

Prescan Normalize Off
Normalize Off
B1 filter Off
Raw filter Off
Elliptical filter On
Mode Inplane
Geometry
Multi-slice mode Interleaved
Series Interleaved
- -
Special sat. Parallel F/H
Gap 10 mm
Thickness 50 mm
- -
- -
Set-n-Go Protocol Off
Table Position H
Table Position 0 mm
Inline Composing Off
- -
Tim CT mode Off
Sequence
Introduction Off
Dimension 2D
Compensate T2 decay Off
Reduce Motion Sens. Off
Contrasts 1
Bandwidth 130 Hz/Px
Flow comp. No
Allowed delay 5 s
Echo spacing 13.1 ms
- -
Define Turbo factor
Turbo factor 7
Echo trains per slice 37
RF pulse type Normal
Gradient mode Fast

T2-weighted:

SIEMENS: tse
Routine
Slice group 1
Slices 14
Dist. factor 40 %
Position Isocenter
Orientation Transversal
Phase enc. dir. R >> L
Rotation 90.00 deg
Phase oversampling 0 %
FoV read 150 mm
FoV phase 100.0 %
Slice thickness 2.2 mm
TR 2500 ms
TE 76 ms
Averages 2
Concatenations 2
Filter Distortion Corr.(2D), Elliptical filter
Coil elements HEA;HEP;NEA;NEP
Contrast
TD 0.0 ms
MTC Off
Magn. preparation None
Flip Angle 120 deg
Fat suppr. SPAIR
Fat sat. mode Strong
Water suppr. None
Restore magn. Off
- -
Averaging mode Long term
Reconstruction Magnitude
Measurements 1
Multiple series Each measurement
Resolution
Base resolution 256
Phase resolution 100 %
Phase partial Fourier Off
Trajectory Cartesian
Interpolation Off
- -
PAT mode None
Matrix Coil Mode Auto (CP)
- -
Image Filter Off
Distortion Corr. On
Mode 2D
Unfiltered images On

Prescan Normalize Off
Normalize Off
B1 filter Off
Raw filter Off
Elliptical filter On
Mode Inplane
Geometry
Multi-slice mode Interleaved
Series Interleaved
- -
Special sat. Parallel F/H
Gap 10 mm
Thickness 50 mm
- -
- -
Set-n-Go Protocol Off
Table Position H
Table Position 0 mm
Inline Composing Off
- -
Tim CT mode Off
Sequence
Introduction Off
Dimension 2D
Compensate T2 decay Off
Reduce Motion Sens. Off
Contrasts 1
Bandwidth 133 Hz/Px
Flow comp. No
Allowed delay 5 s
Echo spacing 15.1 ms
- -
Define Turbo factor
Turbo factor 15
Echo trains per slice 18
RF pulse type Normal
Gradient mode Fast

Proton density-weighted:

SIEMENS: tse
Routine
Slice group 1
Slices 14
Dist. factor 40 %
Position Isocenter
Orientation Transversal
Phase enc. dir. R >> L
Rotation 90.00 deg
Phase oversampling 0 %
FoV read 150 mm
FoV phase 100.0 %
Slice thickness 2.2 mm
TR 2400 ms
TE 16 ms
Averages 2
Concatenations 2
Filter Distortion Corr.(2D), Elliptical filter
Coil elements HEA;HEP;NEA;NEP
Contrast
TD 0.0 ms
MTC Off
Magn. preparation None
Flip Angle 120 deg
Fat suppr. SPAIR
Fat sat. mode Strong
Water suppr. None
Restore magn. Off
- -
Averaging mode Long term
Reconstruction Magnitude
Measurements 1
Multiple series Each measurement
Resolution
Base resolution 384
Phase resolution 100 %
Phase partial Fourier Off
Trajectory Cartesian
Interpolation Off
- -
PAT mode None
Matrix Coil Mode Auto (CP)
- -
Image Filter Off
Distortion Corr. On
Mode 2D
Unfiltered images On

Prescan Normalize Off
Normalize Off
B1 filter Off
Raw filter Off
Elliptical filter On
Mode Inplane
Geometry
Multi-slice mode Interleaved
Series Interleaved
- -
Special sat. Parallel F/H
Gap 10 mm
Thickness 50 mm
- -
- -
Set-n-Go Protocol Off
Table Position H
Table Position 0 mm
Inline Composing Off
- -
Tim CT mode Off
Sequence
Introduction Off
Dimension 2D
Compensate T2 decay Off
Reduce Motion Sens. Off
Contrasts 1
Bandwidth 133 Hz/Px
Flow comp. No
Allowed delay 30 s
Echo spacing 16.1 ms
- -
Define Turbo factor
Turbo factor 15
Echo trains per slice 26
RF pulse type Normal
Gradient mode Fast

Table I Quantitative real-time PCR: primers and probes

Gene	Forward primer (5'-3')	Reverse primer (5'-3')	5' fluorophore	Probe (5'-3')	3' quencher	Amplicon length (bp)
CD68	GGTGGAGTACAATGTGTC	GCTGGTGAAAGAATGATG	FAM	ACGCAGCACAGTGGACATTC	BHQ1	135
IL-18	GCCTCTATTTGAAGATATGAC	CACAGGAGAGAGTTGAAA	Cy5	TTCACAGAGATAGTTACAGCCATACCT	BHQ2	149
CD163	CCTGTGATCACTATGAAGAA	CAGAGAGAAGTCCGAATC	HEX	TCACCATGCTTCACTTCAACACG	BHQ1	146
MMP9	TCCAGTACCGAGAGAAAG	CAGGATGTCATAGGTCAC	FAM	CTGCCAGGACCGCTTCTACT	BHQ1	113
CTSK	AGCCAGACAACAGATTTC	GGTGTCCAGTATCTCCTC	Cy5	TCACCACAGGTAGCAGCAGAA	BHQ2	100
PPIA	GGGTGTTCACAGATCTTG	ACCTCTAAATGCATGAACTC	FAM	AACCTCTTAATTCTGGCTCCTTGCTT	BHQ1	128
RPLP	ACCCTGAAGTGCTTGATA	GAAGGTGTAATCCGTCTC	Cy5	CTCTGCATTCTCGCTTCCTGGA	BHQ2	134
TNFα	GAGGACGAACATCCAACC	GGTCTTGTTGCTTAAAGTTCTA	FAM	TGCCCCAATCCCTTTATTACCCC	BHQ1	150

BHQ = black hole quencher; CD68 = cluster of differentiation 68; CD163 = cluster of differentiation 163; CTSK = cathepsin K; Cy5 = cyanine; FAM = Fluorescein; HEX = hexachlorofluorescein; IL-18 = interleukin 18; MMP9 = matrix metalloproteinase 9; PPIA = peptidylprolyl isomerase A; RPLP = ribosomal protein large P; TNFα = tumor necrosis factor α.

Table II Optimized filter settings, primer and probe concentrations for Taqman® assays on the Mx3000P and Mx3005P systems

Gene	FP concentration (nM)	RP concentration (nM)	Probe concentration (nM)	Filter settings
CD68	300	300	150	FAM x 4
IL-18	300	600	200	Cy5 x 2
CD163	300	300	200	HEX x 1
MMP9	300	300	250	FAM x 4
CTSK	300	300	250	Cy5 x 2
PPIA	300	300	200	FAM x 4
RPLP	300	300	200	Cy5 x 2
TNFα	300	300	300	FAM x 2

CD68 = cluster of differentiation 68; CD163 = cluster of differentiation 163; CTSK = cathepsin K; Cy5 = cyanine; FAM = fluorescein; FP = forward primer; HEX = hexachlorofluorescein; IL-18 = interleukin 18; MMP9 = matrix metalloproteinase 9; PPIA = peptidylprolyl isomerase A; RP = reverse primer; RPLP = 60S acidic ribosomal protein P0; TNFα = tumor necrosis factor α.
